# Characteristics and Outcomes of Immunocompromised Patients With COVID-19 Infection Admitted to an Intensive Care Unit: A Retrospective Cohort Study

**DOI:** 10.7759/cureus.92961

**Published:** 2025-09-22

**Authors:** Xizi Duo, Kush Deshpande

**Affiliations:** 1 Intensive Care Unit, Royal Prince Alfred Hospital, Sydney, AUS; 2 Department of Intensive Care, St. George Hospital, Sydney, AUS; 3 School of Medicine, University of New South Wales, Sydney, AUS

**Keywords:** covid-19, icu patients, immunocompromised, mortality, propensity score matching

## Abstract

Background

Several studies have reported that the course of illness is different in immunocompromised and non-immunocompromised patients with COVID-19 infection. The impact of COVID-19 on immunocompromised patients is not clear due to conflicting evidence from different observational studies.

Aim

This study aimed to compare the characteristics and outcomes of immunocompromised and non-immunocompromised patients with COVID-19 infection admitted to an intensive care unit and to evaluate whether the immunocompromised status is associated with increased mortality.

Methods

We conducted this retrospective cohort study using an electronic database in St. George Hospital, a tertiary ICU in Sydney. We included all adult patients (age >16 years) admitted to the ICU with COVID-19 infection over a 33-month period (March 1, 2020, to November 30, 2022). We collected the data on demographics, comorbidities, clinical characteristics, interventions, and outcomes for all patients. We used logistic regression and multivariate adaptive regression splines (MARS) to determine the predictors of mortality. We used propensity score analyses to check whether immunocompromised patients had higher hospital mortality compared to non-immunocompromised patients.

Results

A total of 258 patients (mean age 61 ± 16 years, males 65%, mean APACHE II score 14 ± 5, unvaccinated 58%, and immunocompromised 17%) were studied. The immunocompromised patients were older, had higher APACHE II and III scores, and had a higher vaccination rate. The hospital mortality was higher in immunocompromised patients (39.5% vs. 14.4%, p < 0.001). On multivariate logistic regression, age (OR 1.05, 95% CI 1.01-1.1, p = 0.02), APACHE III (OR 1.05, 95% CI 1.02-1.09, p < 0.001), vasopressor use (OR 6.7, 95% CI 2.6-17.2, p < 0.001), and single-dose vaccination (OR 3.76, 95% CI 1.19-11.9, p = 0.02) were independent predictors of mortality. APACHE III (score > 70) was the only variable of importance using the MARS model. The "covariate balancing propensity score" analysis did not reveal increased mortality in immunocompromised patients (OR 2.02, 95% CI 0.78-5.23, p = 0.14).

Conclusion

In this small, single-center study, an immunocompromised state was not an independent predictor of hospital mortality. The prediction models for risk of death indicated a trend towards increased mortality.

## Introduction

The COVID-19 pandemic has posed unprecedented challenges to global healthcare systems, with varying outcomes observed across different patient populations. Among the most vulnerable are immunocompromised individuals, who may have heightened susceptibility to severe illness and complications from viral infections. COVID-19 manifests with a wide range of symptoms, including fever, cough, shortness of breath, fatigue, and loss of taste or smell, but can escalate to more severe complications such as pneumonia, acute respiratory distress syndrome (ARDS), multi-organ failure, and death. This variability in clinical presentation underscores the complexity of the disease, particularly in individuals with altered immune responses [1].

Immunocompromised populations are heterogeneous and include individuals with malignancies, organ transplants, autoimmune diseases, or those on chronic immunosuppressive therapies. While it is widely assumed that these individuals face worse outcomes, the literature presents conflicting evidence. For instance, Li et al. found that moderately to severely immunocompromised patients had higher nasal viral loads and greater plasma viremia, suggesting more severe viral dynamics and delayed clearance [2]. Similarly, Gupta et al. showed increased 28-day mortality in ICU patients with active cancer, and Han et al. reported a 44% increased mortality risk and 49% greater risk of severe disease in cancer patients across multiple studies [3,4].

By contrast, other studies found no significant mortality difference after adjusting for confounding factors such as age and comorbidities. These inconsistencies may reflect differences in the type and degree of immunosuppression and underlying illness. For example, Cravedi et al. found that compared to reported mortality rates of the general population requiring hospitalization for COVID-19, the mortality rate of their kidney transplant recipients who needed to be hospitalized with COVID-19 was similar [5]. This highlights how certain forms of immunosuppression might not universally lead to poorer outcomes.

The management of critically ill patients in the ICU presents numerous challenges, including limited resources, staffing shortages, and the need for invasive interventions such as mechanical ventilation and renal replacement therapy. These challenges are compounded in immunocompromised patients, who may require more aggressive monitoring and tailored therapeutic approaches due to their unique clinical profiles.

Understanding the range of symptoms, complications, and specific difficulties encountered in ICU settings for these two groups is crucial for optimizing care strategies and resource allocation. This retrospective cohort study aims to compare the clinical outcomes of immunocompromised and non-immunocompromised patients admitted to our ICU during the COVID-19 pandemic. We hypothesized that the immunocompromised patients with COVID-19 would have higher mortality.

## Materials and methods

Study design

We conducted a single-centre, retrospective cohort study in the ICU of St. George Hospital in Sydney using an electronic database. We included all adult patients (age >18 years) admitted to the ICU who had positive COVID-19 polymerase chain reaction (PCR) results over a 33-month period between March 1, 2020, and November 30, 2022. We excluded patients who had incomplete data due to being transferred to a different unit, as well as patients with incidental COVID-19 infection, which we defined by having no oxygen requirement or any infiltrates on chest X-ray. The records for 258 patients were reviewed, including 43 immunocompromised patients and 215 non-immunocompromised patients. We collected the data on demographics, comorbidities, severity of illness scores including Acute Physiology and Chronic Health Evaluation (APACHE) II [6] and III [7], interventions, and outcome measures including length of stay (LOS) and hospital mortality, for all patients. The primary outcome was hospital mortality. We also compared other outcomes, such as ICU length of stay, number of patients who needed mechanical ventilation and its duration, number of patients requiring vasopressors, and continuous renal replacement therapy (CRRT) in the immunocompromised and non-immunocompromised patients. 

We defined the immunocompromised state using the Australian and New Zealand Intensive Care Society (ANZICS) definitions (Table 1). 

**Table 1:** Immunocompromise definitions

Immunosuppressive disease	Immunosuppressive therapy
Leukemia	Immunosuppression such as in organ transplant
Acquired immunodeficiency syndrome (AIDS)	Chemotherapy
Lymphoma	Radiation therapy
Severe autoimmune disease	High-dose steroids equivalent to >1.5 mg/kg m ethylprednisone for five days or more
Documented metastatic cancer	

Ethics statement

This study was submitted to the Human Research Ethics Committee for the South Eastern Sydney Local Health District (SESLHD) for review and was deemed not to raise any ethical risks requiring submission to an ethical review committee in accordance with NSW Health Policy.

Statistical analysis

We analysed the continuous variables using mean ± SD or median (IQR) and the categorical variables as numbers and percentages (%). To compare the distribution of characteristics and outcomes between immunocompromised and non-immunocompromised patients, we used the Chi-square test or Fisher's exact test for categorical variables and the independent-samples t-test or the non-parametric Wilcoxon Mann-Whitney U test for continuous variables.

We used a multiple logistic regression model and a multivariate adaptive regression splines (MARS) model to determine the predictors of mortality. All variables were included in the logistic regression model. Variables were selected using the "step" function from the "MASS" package in R. This method uses Akaike Information Criteria (AIC) iteratively to select the variables and avoids overfitting and multicollinearity. The MARS model was developed using the "earth" package in R. This method addresses the issues of nonlinearity and collinearity.

We used propensity score analyses [8] to check whether immunocompromised patients had higher mortality compared to non-immunocompromised patients. Covariate balancing propensity score matching was performed using the "cobalt" package in R. All variables were included in the model, and covariate balance was assessed by "absolute standardized mean differences" and "Kolmogorov-Smirnov statistics."

A p-value of <0.05 was considered statistically significant. All analyses were performed using R statistical software (version 4.2.3).

## Results

**Figure 1:**
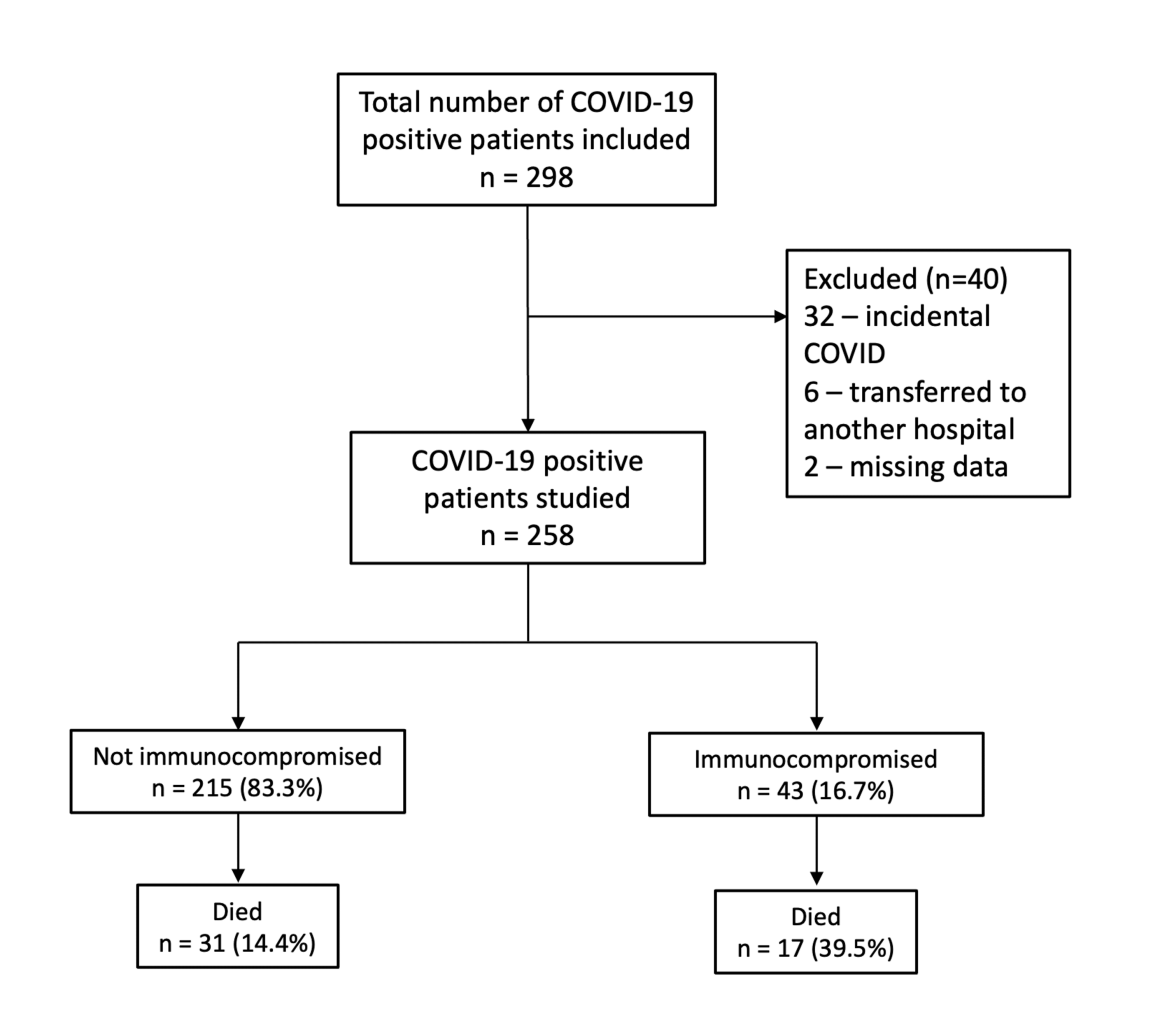
Participant flowchart

The flow of participants in the study is shown in Figure 1, and their baseline characteristics are shown in Table 2. Table 3 shows the underlying conditions resulting in an immunocompromised state. A total of 258 patients (mean age: 61 ± 16 years; males: 65%) admitted to the ICU with COVID-19 were included in this study. Among them, 43 (16.7%) were immunocompromised. The immunocompromised patients were older (mean age: 65.3 ± 12.2 vs. 59.8 ± 16.7 years, p = 0.04), had higher APACHE II (15.6 ± 4.5 vs. 13.7 ± 5.0, p = 0.02) and APACHE III scores (72.6 ± 19.4 vs. 50.5 ± 20.1, p < 0.001), and had higher vaccination rate (79.1% vs. 34.4%, p < 0.001). Table 4 shows the baseline characteristics stratified by the immune status.

**Table 2:** Baseline characteristics of all patients ± indicates mean and SD, *indicates median and IQR, (   ) indicates %, APACHE: Acute Physiology and Chronic Health Evaluation, MV: mechanical ventilation, CRRT: continuous renal replacement therapy, LOS: length of stay

	n = 258
Age, years	60.7 ± 16.2
Gender (Males)	167 (64.7)
APACHE II	14.1 ± 5.0
APACHE III	54.1 ± 21.6
Vaccination	108 (41.9)
Vaccine doses	
0	150 (58.1)
1	28 (10.9)
2	55 (21.3)
3	16 (6.2)
4	9 (3.5)
Immune compromised	43 (16.7)
Respiratory failure	4 (1.6)
Heart failure	2 (0.8)
Cirrhosis	5 (1.9)
Dialysis	2 (0.8)
Non-invasive ventilation	55 (21.3)
Invasive ventilation	100 (38.8)
Vasopressors	105 (40.7)
CRRT	10 (3.9)
MV duration, hours*	0 (0-186)
ICU LOS, days*	5.8 (3.1-12.2)
Hospital LOS, days*	15.2 (8.9-24.7)
Hospital mortality	48 (18.6)
Discharge destination	
Died	48 (18.5)
Home	186 (71.5)
Other hospital	8 (3.1)
Rehabilitation ward	18 (6.9)

**Table 3:** Underlying conditions for immunocompromised state

Cause of immunocompromise	Total = 43, n (%)	Died = 17, n (%)
Metastatic cancer	10 (23.3)	4 (23.5)
Haematological malignancy	9 (20.9)	5 (29.4)
Stem cell transplant	1 (2.3)	0
Solid organ transplant	14 (32.6)	6 (35.3)
AIDS	2 (4.7)	1 (5.9)
Immunosuppressive therapy for other conditions	7 (16.3)	1 (5.9)

**Table 4:** Baseline characteristics stratified by the immune status ± indicates mean and SD, *indicates median and IQR, (   ) indicates %.

	Not immunocompromised (n = 215)	Immunocompromised (n = 43)	p-value
Age	59.8 ± 16.7	65.3 ± 12.2	0.04
Gender (males)	138 (64.2)	29 (67.4)	0.82
APACHE II	13.7 ± 5.0	15.6 ± 4.5	0.02
APACHE III	50.5 ± 20.1	72.6 ± 19.4	<0.001
Vaccination	74 (34.4)	34 (79.1)	<0.001
Vaccine doses			<0.001
0	141 (65.6)	9 (20.9)	
1	23 (10.7)	5 (11.6)	
2	40 (18.6)	15 (34.9)	
3	9 (4.2)	7 (16.3)	
4	2 (0.9)	7 (16.3)	
Respiratory failure	4 (1.9)	0 (0.0)	0.82
Heart failure	2 (0.9)	0 (0.0)	1.0
Cirrhosis	4 (1.9)	1 (2.3)	1.0
Dialysis	2 (0.9)	0 (0.0)	1.0
Non-invasive ventilation	44 (20.5)	11 (25.6)	0.59
Invasive ventilation	88 (40.9)	12 (27.9)	0.15
Vasopressors	86 (40.0)	19 (44.2)	0.73
CRRT	5 (2.3)	5 (11.6)	0.01
MV duration, hours*	0 (0-199)	0 (0-60.5)	0.11
ICU LOS, days*	5.7 (3.2-12.2)	5.8 (2.8-12.5)	0.68
Hospital LOS, days*	14.3 (8.6-24.5)	19.4 (11-24.9)	0.24
Hospital mortality	31 (14.4)	17 (39.5)	<0.001

The hospital mortality rate was significantly higher in immunocompromised patients (39.5% vs. 14.4%, p < 0.001). Independent predictors of mortality identified through multivariate logistic regression included age (OR 1.05, 95% CI 1.01-1.1, p = 0.02), APACHE III score (OR 1.05, 95% CI 1.02-1.09, p < 0.001), vasopressor use (OR 6.7, 95% CI 2.6-17.2, p < 0.001), and single-dose vaccination (OR 3.76, 95% CI 1.19-11.9, p = 0.02). The MARS model identified APACHE III (score > 70) as the most significant predictor of hospital mortality.

**Figure 2:**
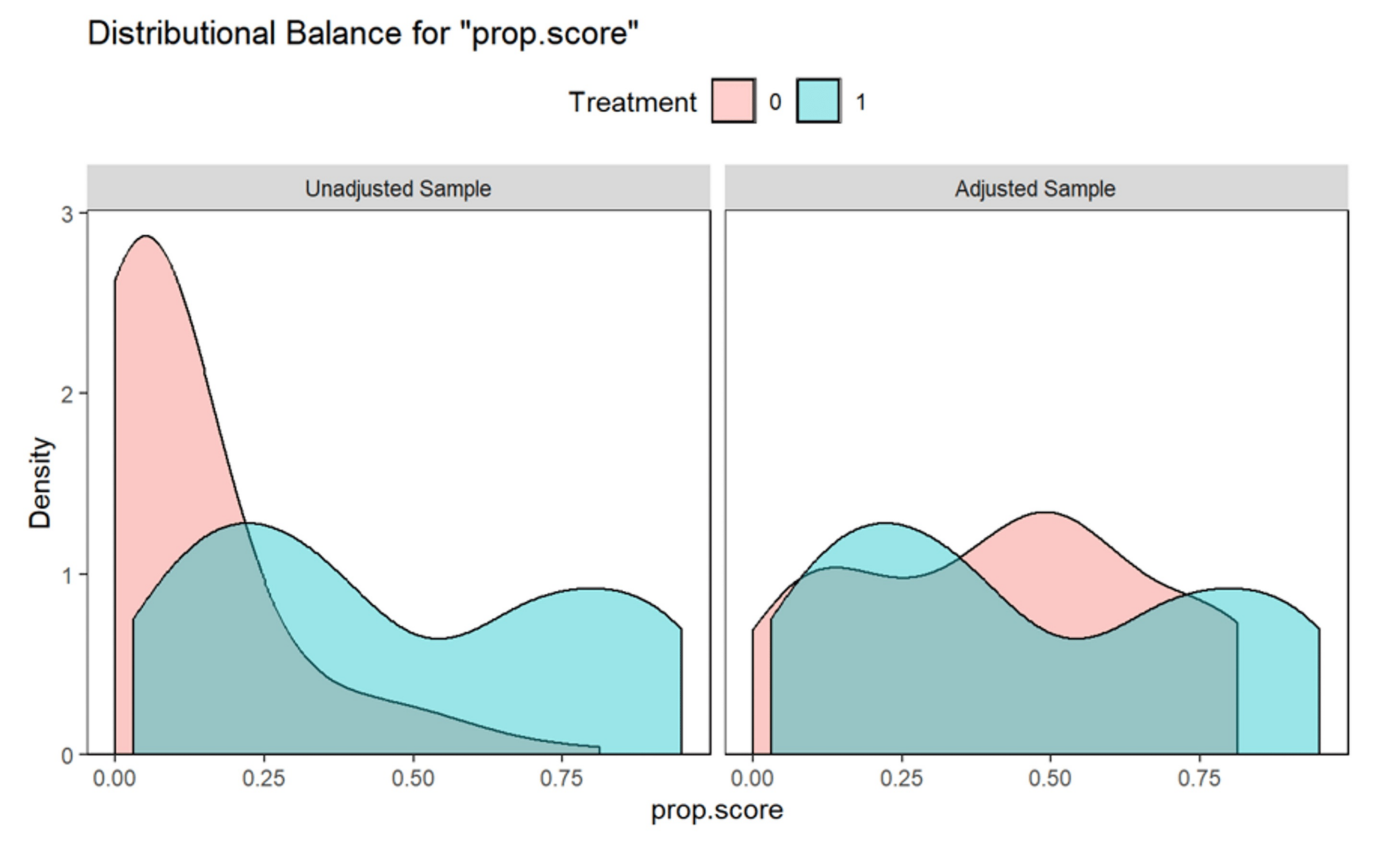
Covariate balancing propensity score

**Figure 3:**
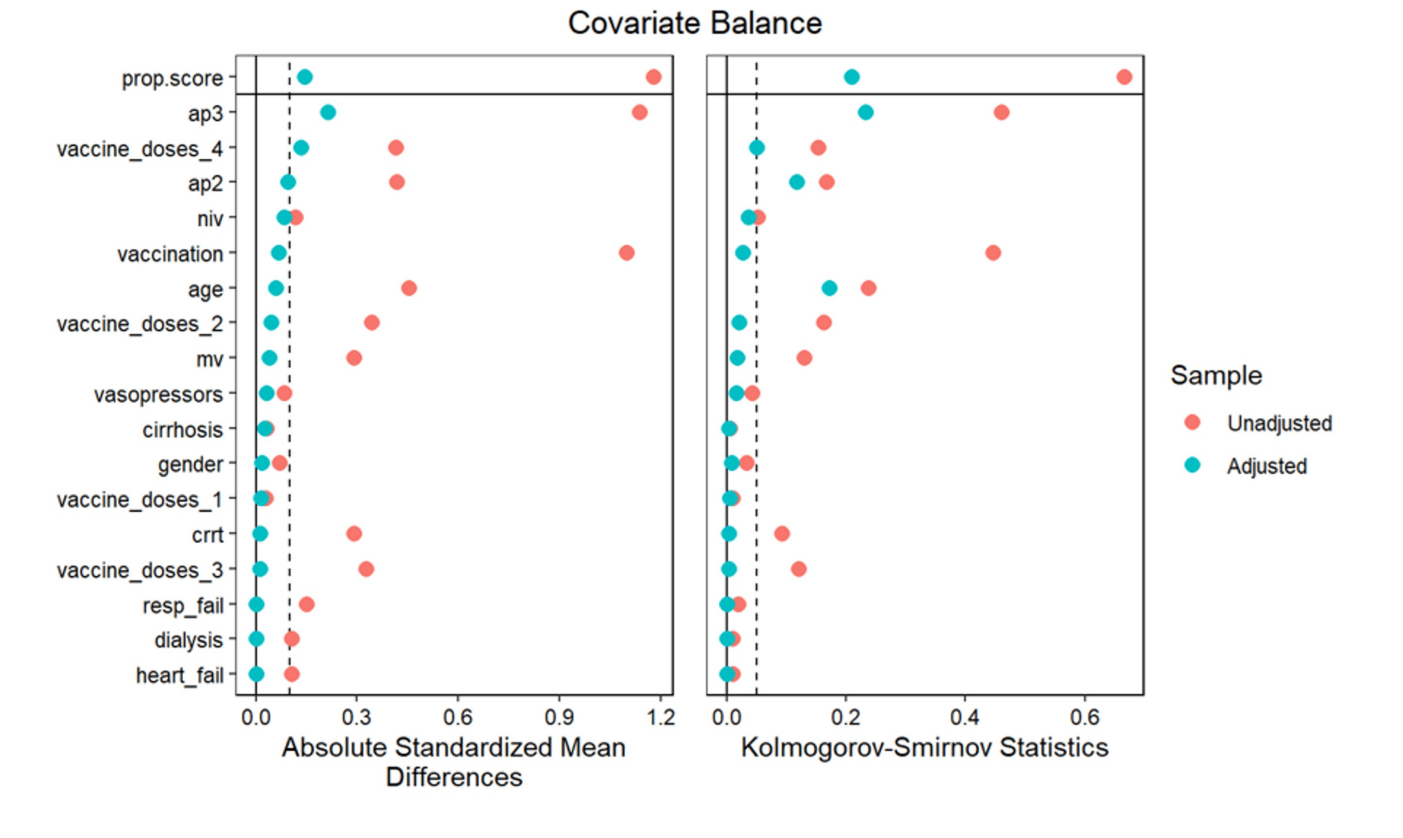
Covariate balancing propensity score by absolute standardized mean differences and Kolmogorov-Smirnov statistics

Figures 2-3 show the matching between the immunocompromised and non-immunocompromised groups using the covariate balancing propensity score method.

Covariate balancing propensity score

The covariate balancing propensity score analysis did not reveal a statistically significant increase in the risk of hospital mortality for immunocompromised patients (OR 2.02, 95% CI 0.78-5.23, p = 0.14), although there was a trend toward higher mortality (Table 5).

**Table 5:** Risk of death in immunocompromised patients *Variables in the multivariate logistic regression model: age, gender, vaccination, vaccine doses, APACHE II and III, NIV, MV, vasopressors, CRRT, respiratory failure, heart failure, dialysis, cirrhosis, immune status

	Odds ratio	95% CI	P-value
Logistic regression (univariate)	3.88	1.89 - 7.97	0.00
Logistic regression (multivariate)	2.97	0.93 - 9.54	0.07
Covariate balancing propensity score matching	2.02	0.78 - 5.23	0.15

## Discussion

Statement of principal findings

This study found that immunocompromised patients admitted to the ICU with COVID-19 had higher severity of illness scores and a significantly increased crude hospital mortality rate compared to non-immunocompromised patients. However, when adjusting for confounding factors using multivariate regression and propensity score methods, immunocompromised status was not identified as an independent predictor of hospital mortality. Instead, age, severity of illness (APACHE III score), vasopressor use, and single-dose vaccination were significant predictors of hospital mortality.

Comparison with similar studies

Previous studies have presented mixed results regarding the impact of immunocompromise on COVID-19 outcomes. Some meta-analyses, such as Belsky et al. [1], found that only solid organ transplant patients had a significantly increased risk of hospital mortality compared to non-immunocompromised patients, whereas cancer and hematopoietic cell transplant patients did not show statistically significant differences. Similarly, Gatti et al. and Raja et al. found no statistically significant difference in mortality for solid organ transplant patients compared to non-immunocompromised patients, suggesting that immunocompromised status alone may not be a determinant of worse outcomes [9,10].

Conversely, retrospective cohort studies, such as those by Suárez-García et al. in Spain and Baek et al. in Korea, reported significantly higher mortality in immunocompromised patients compared to their non-immunocompromised counterparts [11,12]. Specifically, Suárez-García et al. found an adjusted odds ratio (aOR) of 1.6 (95% CI 1.43-1.79), with transplant patients showing the highest risk [8]. Likewise, the WHO ISARIC CCP-UK study found an increased risk of mortality among immunocompromised patients (aOR 1.44, 95% CI 1.39-1.50, p < 0.001) [13].

By contrast, a large retrospective cohort study using the National COVID Cohort Collaborative found no significant association between immunosuppression and in-hospital mortality after propensity score matching, except for patients receiving rituximab for rheumatologic or cancer-related conditions [14]. Andersen et al. similarly found that rituximab had worse outcomes; however, not all immunosuppressive medications had a significant association between immunosuppression and worse outcomes [15]. 

Our findings align more closely with studies that emphasize the importance of overall illness severity rather than immunocompromised status alone. The strong influence of APACHE III scores and vasopressor use on hospital mortality in our study suggests that clinical factors beyond immune status may play a more significant role in predicting outcomes in critically ill COVID-19 patients.

The heterogeneity of immunocompromised states remains a critical challenge in interpretation. In our study, subgroup numbers were too small to differentiate between transplant recipients, autoimmune conditions, and malignancy.

Importantly, a meta-analysis by Han et al. emphasized cancer as a distinct high-risk group, but did not find consistent outcome differences in other forms of immunosuppression [4]. This supports the idea that future studies should stratify by immunosuppression type and intensity, ideally in multicenter or pooled cohorts.

Our study not only contributes to the growing body of literature on COVID-19 but also highlights the need for tailored approaches in managing critically ill patients with varying immune profiles, ultimately aiming to improve outcomes for the most at-risk populations.

Strengths and limitations

One of the strengths of this study is the well-defined cohort with accurate and comprehensive data collection. The use of robust statistical analysis methods, including logistic regression, MARS modelling, and propensity score analysis, strengthens the reliability of the findings by accounting for potential confounders. In addition, this study contributes valuable data from a tertiary ICU in Australia, a region where limited research has been published on immunocompromised COVID-19 patients. The study’s high internal validity is another key strength, enhancing the credibility of the results.

However, there are several limitations. The single-center, retrospective study design limits the generalizability of the findings. The small sample size, particularly the limited number of immunocompromised patients and their subgroups, restricts the ability to draw definitive conclusions about specific immunocompromised conditions. Patients who were transferred to other hospitals were not included, which may have introduced selection bias. Another limitation is the lack of data on different SARS-CoV-2 variants, which may have influenced disease severity and outcomes. Additionally, immunocompromised patients constitute a heterogeneous group with varying degrees of immune dysfunction, which may impact outcomes.

Implications for future research

Given these findings and the conflicting evidence in the literature, a larger multicentre study is needed to further evaluate the impact of immunocompromised status on COVID-19 ICU outcomes. Future research should also consider subgroup analyses based on specific causes of immunosuppression, as different immunocompromised conditions may have varying risks and prognoses.

## Conclusions

This study did not find immunocompromised status to be an independent predictor of mortality in our ICU cohort of COVID-19 patients. While the mortality rate was higher among immunocompromised individuals in unadjusted analyses, this association did not hold in multivariate analyses or propensity score assessments. There is also a need for subgroups of immunocompromised patients to be studied separately. The immunocompromised patients are a heterogeneous group and have varying degrees of mortality with COVID-19. Further studies with larger sample sizes and multi-center designs are necessary to explore these findings in greater detail.
